# In Vitro Selection of Specific DNA Aptamers Against the Anti-Coagulant Dabigatran Etexilate

**DOI:** 10.1038/s41598-018-31327-3

**Published:** 2018-09-05

**Authors:** Maher M. Aljohani, Raja Chinnappan, Shimaa Eissa, Omar A. Alsager, Karina Weber, Dana Cialla-May, Jürgen Popp, Mohammed Zourob

**Affiliations:** 1 Department of Chemistry, Alfaisal University, Al Zahrawi Street, Al Maather, Al Takhassusi Rd, Riyadh, 11533 Saudi Arabia; 2 Collage of Medicine, Taibah University, Madinah, Saudi Arabia; 3 InfectoGnostics Research Campus Jena, Center for Applied Research, Friedrich-Schiller-University, Philosophenweg7, Jena, 07743 Germany; 4 King Abdulaziz City for Science and Technology (KACST), P.O Box 6086, Riyadh, 11442 Saudi Arabia; 5 Institute of Physical Chemistry and Abbe Center of Photonics, Friedrich Schiller University Jena, Helmholtzweg 4, 07743 Jena, Germany; 6 Leibniz Institute of Photonic Technology, Albert-Einstein-Straße 9, 07745 Jena, Germany; 7 King Faisal Specialist Hospital and Research Center, Zahrawi Street, Al Maather, Riyadh, 12713 Saudi Arabia

## Abstract

Dabigatran Etexilate (PRADAXA) is a new oral anticoagulant increasingly used for a number of blood thrombosis conditions, prevention of strokes and systemic emboli among patients with atrial fibrillation. It provides safe and adequate anticoagulation for prevention and treatment of thrombus in several clinical settings. However, anticoagulation therapy can be associated with an increased risk of bleeding. There is a lack of specific laboratory tests to determine the level of this drug in blood. This is considered the most important obstacles of using this medication, particularly for patients with trauma, drug toxicity, in urgent need for surgical interventions or uncontrolled bleeding. In this work, we performed Systematic evolution of ligands by exponential enrichment (SELEX) to select specific DNA aptamers against dabigatran etexilate. Following multiple rounds of selection and enrichment with a randomized 60-mer DNA library, specific DNA aptamers for dabigatran were selected. We investigated the affinity and specificity of generated aptamers to the drug showing dissociation constants (K_d_) ranging from 46.8–208 nM. The most sensitive aptamer sequence was selected and applied in an electrochemical biosensor to successfully achieve 0. 01 ng/ml level of detection of the target drug. With further improvement of the assay and optimization, these aptamers would replace conventional antibodies for developing detection assays in the near future.

## Introduction

**Figure 1:**
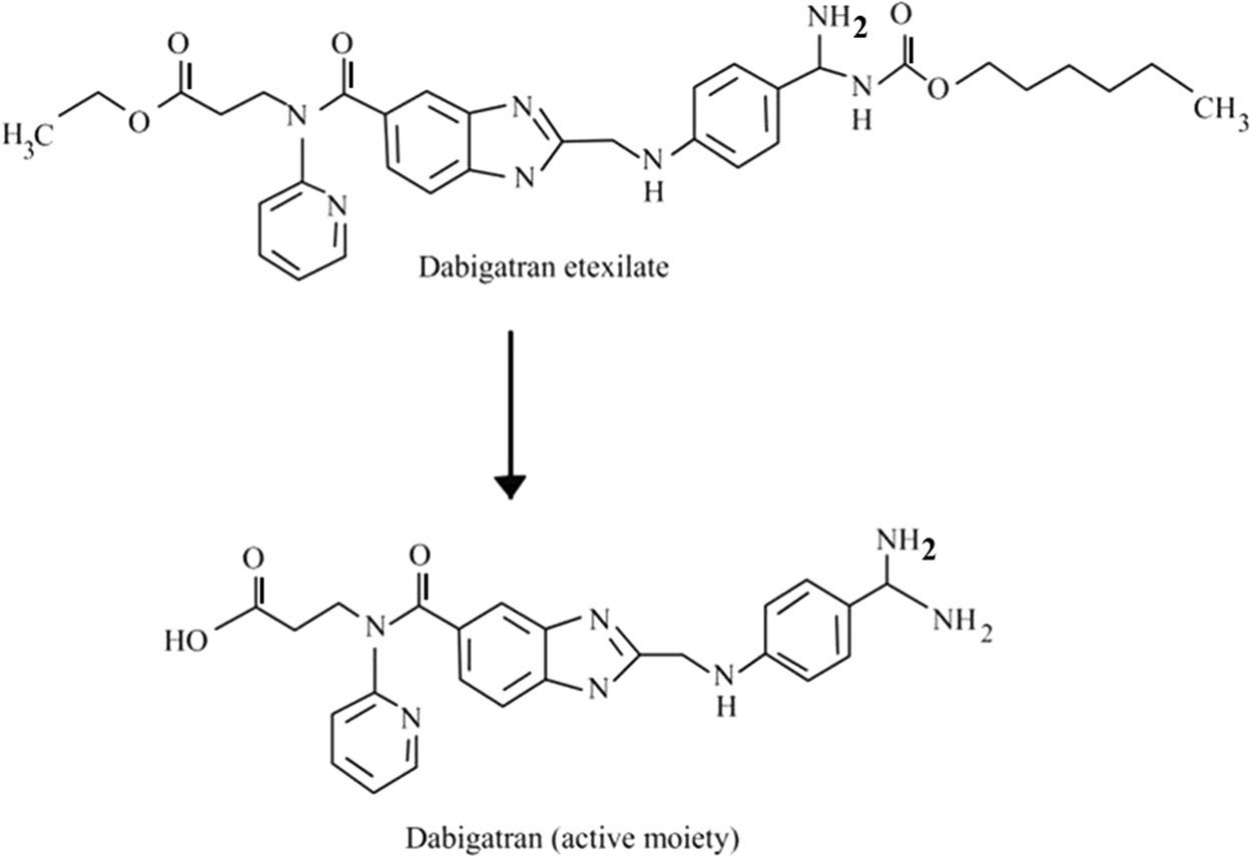
Structure of dabigatran etexilite, a synthetic oral prodrug that is converted to active moiety for the anti-coagulant drug, dabigatran by a serum esterase.

The increase in the thromboembolism disorders, including myocardial infarction, stroke, and deep vein thrombosis affects different sites of the body which leads to disability and death around the globe^1,2^. A wide variety of inherited, acquired or combinations of risk factors result in an increased incidence of thromboembolism. The presence and persistence of these risk factors can be used to predict the incidence and recurrence of thromboembolis^3^. Treatment goals for thromboembolism disorders take account of stopping clot propagation and preventing the recurrence of a new thrombus. Warfarin (Coumadin) is the landmark agent for anticoagulation and prevention of thromboembolism^4^. For several decades warfarin therapy was the only oral anticoagulant treatment for various conditions that requires anticoagulation. Warfarin’s limitations have stimulated scientific interest to develop alternative anticoagulants drugs with fewer shortcomings^5^. Several new oral anticoagulants (NOACs) that are more effective at targeting the mechanism of blood coagulation and blood clot formation have been developed. Dabigatran etexilite is a synthetic oral prodrug that is converted by a serum esterase to dabigatran (Fig. 1). It is a potent, direct and competitive reversible thrombin inhibitor^6^. By binding to the thrombin enzymatic active site via ionic interactions, dabigatran is capable of rapidly and reversibly inhibit both free and clot-bound thrombin and clot formation^7,8^. Dabigatran, sold under the brand name Pradaxa, was first approved by the United States Food and Drug Administration (USFDA) in October 2010, and subsequently in more than 100 countries. Dabigatran is a promising oral anticoagulant because of its rapid onset of action, lack of interaction with other food and drugs, good safety profile, and fixed-dose administration. Dabigatran has also a wider therapeutic window compared to warfarin^8^.

Dabigatran has short half-life (7–17 h) in patients with adequate renal function. Instances of minor bleeding in patients using dabigatran can be managed by stopping the drug until its effects are reversed by clearance. Patients experienced renal disease, have impaired drug clearance and dabigatran half-life can be up to 3-fold^9^. Dabigatran therapy carries a risk of life-threatening bleeding that typically occurs after renal impairment. Unfortunately, in cases of massive bleeding or emergency surgery, no specific laboratory test exists to detect and identify the concentration of this drug. Identifying the toxicity of dabigatran using coagulation assessment by conventional clinical laboratory tests (like the International Normalized Ratio, INR) coagulation test used for warfarin) is inaccurate in the event of a potential overdose or severe life-threatening bleeding. Determination of the therapeutic levels of dabigatran is needed in an emergency, life-threatening situations such as acute massive bleeding and thrombosis, or in the case of emergency surgery^10^. The analysis may also be needed in situations where reversal of dabigatran is medically necessary such as before using the new reversing agent PRAXBIND^11,12^.

Dabigatran can interfere with most of the clot-based coagulation assays that are used to assess blood clotting function in patients, such as the prothrombin time (PT), activated partial thromboplastin time (aPTT), the INR, and thrombin time (TT) tests^13^. Moreover, the standard coagulation assays are not sensitive enough to ensure a quantitative measurement of dabigatran. The results of standard coagulation assays are not reliable and are highly variable in treated patients^13^. The TT assay is a blood test designed to assess the hemostasis step of the conversion of fibrinogen to fibrin and blood clot formation. A modification of the TT assay by patient plasma dilution in pooled normal plasma led to the development of the diluted thrombin time (DTT) method. The DTT method is reliable and indirectly detects the anticoagulation effects of dabigatran at low-to-intermediate levels (dabigatran concentration ≤100 µg/L). However, it is not reliable if dabigatran is present in high or toxic levels^14^.

Liquid chromatography-tandem mass spectrometry (LC*-*MS*/*MS) can be used to measure dabigatran over a broad concentration range accurately. This technology enables the user to measure dabigatran levels in plasma^15^, but it is available only in a few highly specialized laboratories, requires labor-intensive protocols, involves slow turnaround times, and is not available all the time. Thus, LC*-*MS*/*MS is ineffective for rapid testing in clinical laboratories or emergency rooms^16^. Therefore, there is an urgent need for rapid and sensitive detection methods for the detection of dabigatran at the point of care.

Dabigatran is a low molecular weight compound that induce an immune response only when attached to immunogenic conjugates and carrier proteins. *Thus*, *for producing a monoclonal antibody against* dabigatran, a carrier protein has to be used. Idarucizumab (Praxbind) — is a humanized monoclonal antibody fragment (Fab) that binds dabigatran and has the capacity to reverse its anticoagulant effect. The affinity of idarucizumab for dabigatran is more than 300-fold greater than for thrombin^17,18^. Recently, idarucizumab was approved to reverse anticoagulation with dabigatran for the management of emergency situations in dabigatran-treated patients^19^. Idarucizumab is developed from monoclonal antibodies and it can induce adverse body response and serious immunogenic reactions. Dabigatran specific antibody has been also used for the development of an Enzyme-Linked Immunosorbent assay (ELISA) to measure dabigatran concentrations in the range of 7.8 to 125 ng/mL^20^. However, antibody based methods suffer from the instability of the antibodies at harsh conditions and their sophisticated and high production cost.

Over the last years, aptamers have been emerging as promising alternatives to antibodies and were used as a biological recognition element for biosensing, disease diagnosis and therapeutic applications^21–23^. Aptamers have advantages over monoclonal antibody of being greatly specific, stable, relatively small biomolecules, and nonimmunogenic^24^. Aptamers are chemically synthesized with superior stability, can be produced with higher reproducibility and reliability compared to the *in vivo* developed antibodies. Furthermore, aptamers can bind to highly toxic or non-immunogenic antigens that cannot be achieved in animal*-*based methods for antibodies production^25^. To the best of our knowledge, there are no aptamers available for dabigatran etexilite. The aim of this work is to develop specific aptamer for dabigatran etexilite in order to be further integrated in simple sensing platform for the rapid detection and quantification of dabigatran etexilite. This would help to assess and manage dabigatran treated patients in the emergency situations and that can guide the physician for the right decision.

## Results and Discussions

### DNA Aptamer selection

**Figure 2:**
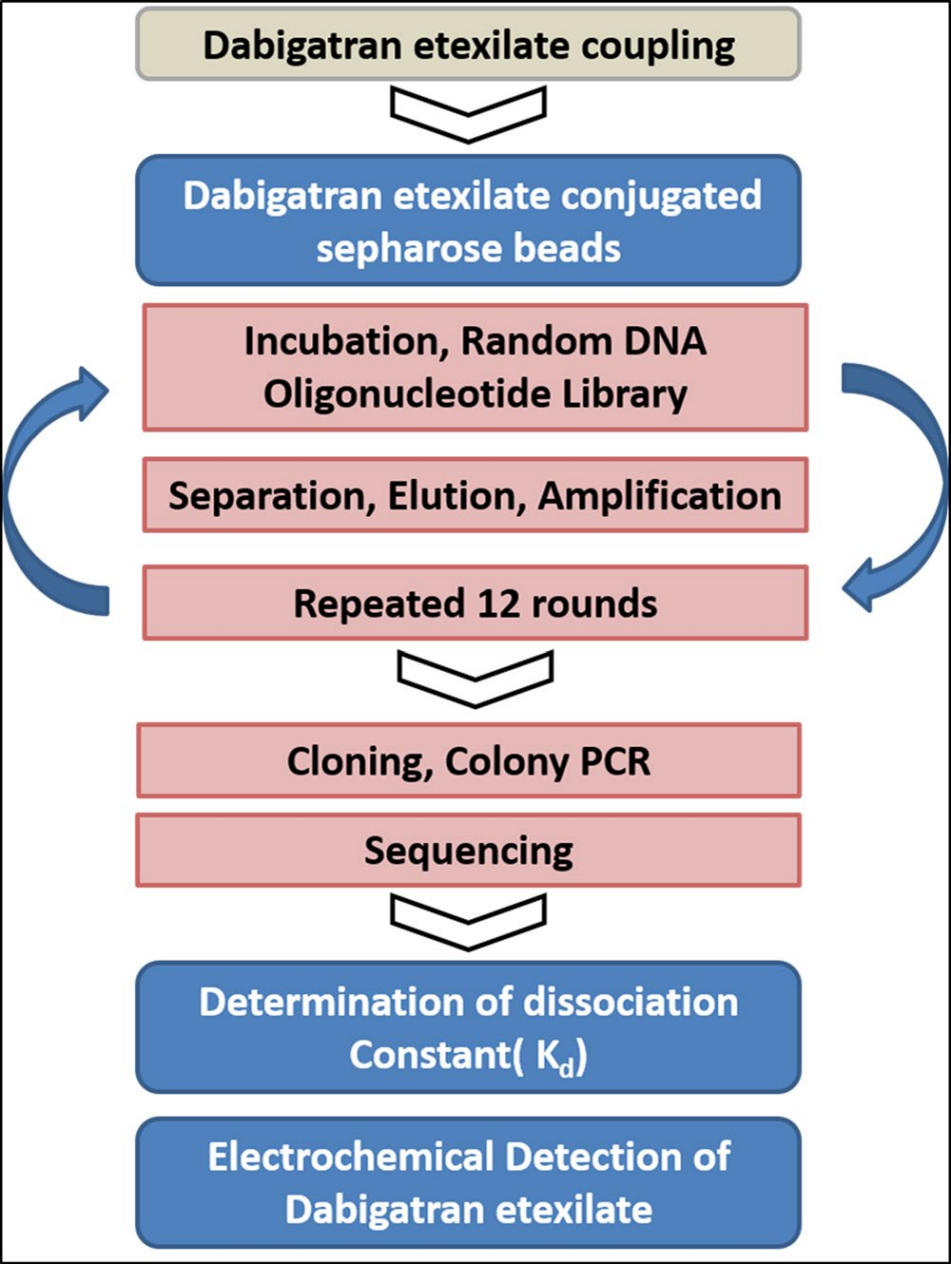
Schematic representation of various processes involved in the ssDNA aptamer selection against dabigatran etexilite.

Here, we report for the first time the identification of ssDNA aptamers targeting dabigatran etexilate, a small anticoagulant drud with a molecular weight of 627.734 g/mol(chemical structure is shown in Fig. 1). The developed aptamers have potential for reversing the anticoagulation activity of dabigatran and enabling significant control over the coagulation process and bleeding risks^26,27^. For SELEX screening, the target molecules are usually immobilized on a solid support surface to facilitate the process of isolating the bound sequences from non-bound sequences. Therefore, dabigatran etexilate was coupled to sepharose beads. The NH_2_-terminal moiety of the molecules is conjugated with NHS activated sepharose beads according to the manufacture. All the other functional groups in the dabigatran etexilite are available for the ssDNA binding during the SELEX process. In the SELEX procedure the ssDNA library pool of 10^15^ random 60 nucleotide sequences were mixed with dabigatran etexilate-beads. The enrichment in the ssDNA binding aptamer was monitored by fluorescence intensity of the eluted ssDNA. The procedure of SELEX process is shown in Fig. 2.

**Figure 3:**
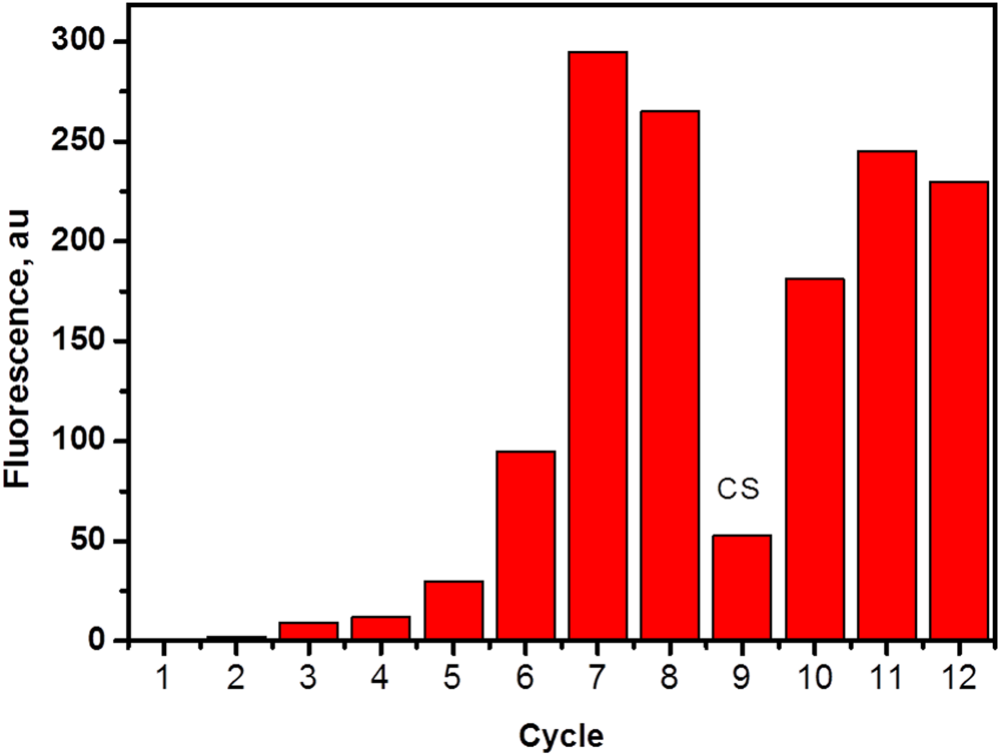
Increase in the number of copies of dabigatran etexilite specific aptamers during the SELEX procedure. The bar graphs represents the fluorescence intensity of the bound ssDNA eluted from Dabigatran etexilite conjugated sepharose beads in each round. The sepharose bound ssDNA was eliminated by counter selection (CS) at round.9.

Figure 3 shows the bar graph of the amount of ssDNA eluted from dabigatran etexilate -sepharose beads in each selection cycle. We found a significant increase of the DNA recovery with each progressive cycle of the selection indicative of the enrichment of dabigatran etexilate binding ssDNA. The counter selection step (CS) was performed in the ninth round to eliminate the non-specifically bound ssDNA to the sepharose beads and a drop in the fluorescence intensity was seen as shown in the Fig. 3. After counter selection, three more rounds of SELEX were done to reach the fluorescence intensity plateau. The constant fluorescence recovery after 10 rounds indicates that the target binding sites are saturated and there are no more sites available for binding. Once the binding amount reached a plateau between consecutive cycles, the pool is considered “enriched” for dabigatran etexilate, the selection cycles were stopped and the DNA was eluted and cloned. 25 clones were picked and sequenced. Four aptamer sequences were successfully obtained as a result of this selection. The four sequences were taken further for binding affinity studies with dabigatran etexilate.

### Determination of the dabigatran aptamers dissociation constants

**Figure 4:**
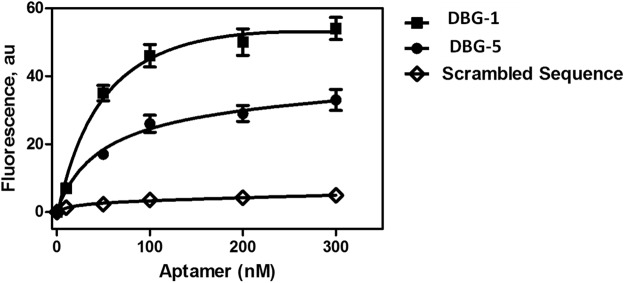
Determination of equilibrium dissociation constant (K_d_) from the saturation binding affinity obtained from the titration of fluorescein labelled aptamers and scrambled ssDNA with constant amount dabigatran etexilite conjugated sepharose beads.The fluorescence spectra were recorded by exciting at 470 ± 10 nm and the emission intensity (515 nm) was used for the plots. The dissociation constants were calculated by nonlinear regression analysis. The error bars represent the standard deviation of three different measurements.

The binding affinity and adhesion strength between the selected aptamers to dabigatran etexilate were studied. The K_d_ values of the selected DNA aptamers were determined using fluorescence-based assay. In this assay, a constant amount of beads were incubated with different concentrations of fluorescein labelled ssDNA aptamer ranging between 0 and 300 nM in the SELEX conditions. After 1 h. of incubation, the unbound aptamers were washed and dabigatran etexilate bound aptamers were eluted by elution buffer. The fluorescence intensity of the each aptamer was plotted against the amount of input aptamer. The K_d_ of each aptamer sequence was obtained from the binding affinity plot by fitting using nonlinear regression analysis (Fig. 4). All the four aptamers showed good affinity to dabigatran etexilate in the nanomolar range from 46.8 to 312 nM (Table 1). Aptamer sequence (DBG-1) and aptamer sequence (DBG-5) showed the highest binding affinity, with K_d_ values of 46.8 nM and 59.6 nM respectively. The binding affinity, K_d_ of 46.8 nM, for the aptamer obtained in this work is comparable to a number of reported aptamers for small molecule^28–30^.

**Table 1:** Dissociation constants (Kd) of the selected aptamers against dabigatran.

Name	K_d_ (nM)
DBG1	46.8
DBG2	208
DBG4	312
DBG5	59.6

### Application of the aptamer in an electrochemical biosensor

**Figure 5:**
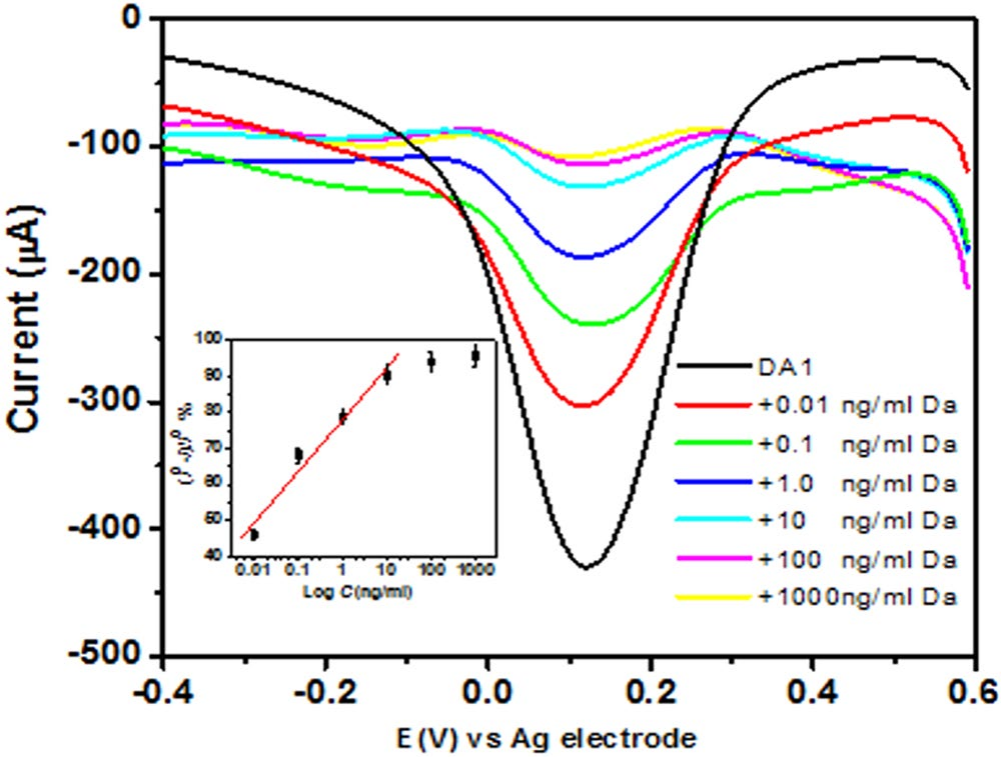
SWV recorded for the aptasensor in 5 mM [Fe(CN)_6_]^3−/4−^ solution prepared in 10 mM PBS, pH 7.4 before and after binding with different concentrations of dabigatran execilite (0 ng/ml, 0. 01 ng/ml, 0.1 ng/ml, 1.0 ng/ml, 10.0 ng/ml, 100.0 ng/ml, 1000.0 ng/ml). Inset is the calibration curve (a plot of the percentage change in the reduction peak current versus the logarithm of the drug concentration).

Based on the K_d_ values from the fluorescence assay, we selected the aptamers with the lowest K_d_ for initial biosensor application (DBG1 and DBG5). The aptamers with the highest affinity to dabigatran etexilate were DBG1 and DBG5. For the aptasensor fabrication, a thiol modified DBG1 aptamer was immobilized on a gold screen printed electrodes via self-assembly. Figure 5 shows the SWV of the aptasensor in ferro/ferricyanide redox couple. It can be clearly seen that a sharp reduction peak of the redox couple was obtained for the aptamer-immobilized electrode. However, after the dabigatran etexilate binding, the reduction peak current decreased likely due to the conformational change of the aptamer upon binding. A more compact target-aptamer conformation is likely to hinder the accessibility of the redox to electrode surface and therefore a sequential reduction of the current is observed upon increasing the target concentration. Evidently, the SWV reduction peak was gradually decreased with increasing the concentration of the analyte which represent the basis of the detection. The inset in the Fig. 5 shows a linear relationship between the logarithm of the dabigatran etexilite concentration and the percentage change in the reduction peak current within the range of 0.01–1000 ng/ml. The resolved analytical window is highly relevant to the potential application of the developed sensor as the bleeding risk increases continuously with dose and there is strong correlation between the rate of major bleeding and plasma concentration of dabigatran etexilate^31^. After approval for dabigatran marketing, more than 9049 reports of dabigatran related bleeding events globally, that includes 368 bleeding related deaths. FDA has approved dabigatran with the 150 mg strength. The dabigatran dose is proposed to reach a therapeutic level from (48–200 ng/mL)^32^. which falls within the range of our detection assay. Therefore, this selected aptamer could be successfully utilized for the detection of dabigatran for point-of-care testing and monitoring.

## Experimental

### Materials and methods

*Dabigatran* etexilate was purchased from (*Selleck*, Houston, TX, USA). N-hydroxysuccinimide (NHS) activated sapharose-4B was purchased from GE healthcare (Milwakee, WI, USA). PBS buffer tablets, Tris base, sodium chloride, sodium acetate, sodium bicarbonate, sodium azide hydrochloric acid, magnesium chloride septavedin and bovine serum aulbamin (BSA), sodium carbonate anhydrous, sodium bicarbonate, sodium azide, dipotassium hydrogen orthophosphate, potassium dihydrogen orthophosphate urea, boric acid, ethanol, dimethyl sulphoxide (DMSO), EDTA disodium dihydrate acrylamide and bisacrylamide, potassium ferrocyanide (K_4_Fe(CN)_6_), potassium ferricyanide (K_3_Fe(CN)_6_), were purchased from Sigma-Aldrich (St Louis, MO, USA). Taq plus DNA polymerase, dntps and 100 base pair ladder were purchased from ACE Biotech (Riyadh, Saudi Arabia). HPLC purified labeled and unlabeled oligonucleotides and random DNA library were purchased from Metabion International (Planegg, Germany). The DNA oligonucleotides were dissolved in double distilled water to make the stock solutions and stored at −20 °C until further use. The DNA solutions used in the experiments were diluted in binding buffer. The fluorescein-labelled oligonucleotides were protected from the light while performing the experiments. Spin-X cellulose acetate centrifuge filter tubes with pore size of 45 µm were obtained from Corning life sciences (Tewksbury, MA USA). 0.5 ml ultrafiltration Amicon device with 3 kDa cutoff desalting filter was purchased from Millipore (Sigma MA USA). pcr2.1-TOPO cloning vector with One Shot MAX Efficiency DH5α-T1 were purchased from Invitrogen Inc, (New York USA). Binding buffer used in this study consists of 50 mM Tris, pH 7.5, 150 mM NaCl and 2 mM MgCl_2_. Elution buffer is 7 M urea in binding buffer. 0.1 M NaHCO_3_ containing 0.5 M NaCl, pH 8.3 was used for the coupling of dabigatran exclite to the NHS activated sepharose beads. 50 mM Tris, pH 8.0, 0.5 M NaCl and 50 mM sodium acetate pH 4.0, 0.5 M NaCl are used for washing the dabigatran exclite conjugated beads. Tris-EDTA buffer (TE) is 10 mM Tris, pH 7.4, 1 mM EDTA. A 10 mM phosphate buffered saline (PBS) solution (pH 7.4) was used for the preparation of the [Fe(CN)_6_]^4−/3−^ redox solution for measurement. All solutions were prepared using Milli-Q grade water.

### Instrumentation

UV-Vis spectrophotometers (NanoDrop 2000C spectrophotometer) used to determine the concentration of the DNA and fluorescence analysis for the fluorescein labelled aptamers were achieved by using Nanodrop ND3300 fluorospectrometer (Thermo Scientific, Canada). The samples were excited in the light emitting diodes (LEDs), λ excitation = 470 ± 10 nm and the emission was monitored at 515 nm. Experiments were performed in triplicate unless otherwise mentioned. All the measurements were recorded in binding buffer (pH 7.4) at room temperature. The fluorescence spectra are the average of two measurements. The Autolab potentiostat/galvanostat instrument (PGSTAT302N, Eco Chemie, The Netherlands) and the Nova (Version 1.11) software are used for the electrochemical experiments. Disposable gold screen printed carbon electrodes were purchased from Dropsens, Inc. (Spain) and used for the electrochemical aptasensor experiments for the specific detection dabigatran etexilate.

### Preparation of Dabigatran etexilate conjugated sapharose beads

Dabigatran etexilate was conjugated with NHS activated sapharose-4B beads according to the manufacturer protocol. Briefly, sapharose-4B beads were washed with 10–15 volumes of 1 mM HCl followed by coupling buffer for 5 times. Then, 100 µl of 10 mg/ml of dabigatran etexilate in DMSO was added to the slurry of the washed sapharose-4B beads and mixed for three hours in 50 mM carbonate buffer (pH = 9.2) at room temperature. The dabigatran etexilate -conjugated beads were washed with carbonate buffer to remove the unreacted dabigatran etexilate. The unreacted active sites in the beads were then quenched by treating them with 50 mM Tris buffer (pH = 8) for one hour. After blocking, the beads were washed with 50 mM Tris, 0.5 M NaCl pH = 8 and 50 mM sodium acetate, 0.5 M NaCl, pH = 4.0–4.5 alternatively for 6 times. The washed beads were stored in 10 mM Tris, pH = 7.5 containing 0.05% sodium azide at 4 °C until further use.

### Library and primer design

A random single-stranded DNA library (3 nmol of 2 × 10^15^ sequences) was used for the SELEX experiments. The PAGE purified library consists of central random region of 60 nucleotides with two fixed regions of 18 nucleotides-sequences at the 3′ and 5′ ends. These regions are the primer binding sites for the polymerase chain reaction (PCR) amplification. The full-length library is (5′-ATACCAGCTTATTCAATT - N60 -AGATAGTAAGTGCAATCT-3′, 96-mer). In order to separate the double strands of the PCR product into two single strands by denaturting PAGE, we design fluorescein labelled forward primer and the reverse primer consists of 18-atom hexa-ethyleneglycol spacer (to block the PCR amplification) followed by polyA_20_. The forward primer is: 5′-fluorescein-ATACCAGCTTATTCAATT-3′ and reverse primer is: 5′-poly dA20-HEGL-AGATTGCACTTACTATCT-3′. After the completion of SELEX rounds, unlabeled primers are used for PCR amplification of the last round DNA for cloning.

### *In Vitro* Selection

The selection process was started by washing 100 µl of Dabigatran-conjugated saparose beads for 5 times with 500 μL binding buffer 150 pmol ssDNA library pool (3 nmol for first round) were used. After heating at 90 °C for 5 min, the solution was cooled to 4 °C for 10 min and then at room temperature (RT) for 5 min and then added to the washed dabigatran etexilate beads. The mixture was incubated at room temperature with end-over-end rotation for 2 h, followed by washing with binding buffer until no fluorescent DNA is detected in the washes. DNA oligonucleotides bound to Dabigatran etexilate beads were eluted with 300 μL aliquots of elution buffer (7 M urea in binding buffer) and heated for 10 min at 90 °C until no fluorescence is detected. Eluted DNA was concentrated and desalted by ultra-filtration device with a 3 kDa cutoff membrane. The negative/counter selection was performed at round number 9against blank sepharose beads (i.e., blocked sepharose beads without conjugated dabigatran). Desalted DNA pool were collected in this counter selection step, subjected to the same heating and cooling treatment as above and subsequently incubated with dabigatran beads.

The selected DNA pools were amplified by polymerase chain reaction (PCR) in 15 parallel 75 μL reactions each containing 2 units of Taq Plus polymerase, buffer, 2 mM MgCl_2_, 200 μM dNTP, 0.2 μM of forward and reverse primer. PCR conditions used in this study were as follows: 94 °C for 10 min, followed by 25 cycles of 94 °C for 1 min, 47 °C for 1 min, 72 °C for 1 min, and a final extension step of 10 min at 72 °C. PCR products were dried by lyophilizer and resuspended in 50:50 water and formamide and heated to 90 °C for 5 min. The relevant DNA strand labeled with fluorescein and PolyA were separated from the double stranded PCR product in 10% denaturing PAGE. The fluorescence ssDNA band from the gel was sliced and eluted by freeze−thaw cycle. Eluted ssDNA in TE (10 mM Tris, pH 7.4, 1 mM EDTA) was concentrated by ultrafiltration and used for the next round of selection.

### Cloning and sequencing of the selected DNA aptamers

After twelve rounds of SELEX, the DNA recoveries begin to plateau that were monitored by fluorescence intensity of the eluted DNA. The selected ssDNA from the last round were amplified with the un-modified primer set and cloned into pCR2.1-TOPO vector using the TOPO TA Cloning Kit. Colonies were grown on Lauria *Broth* (LB) *agar* medium supplemented with ampicillin, X-Gal. Positive colonies)white or light blue colonies (were picked and grown in liquid media. ssDNA inserts were PCR amplified using M13 forward and reverse primers. PCR products were sequenced and aligned by using *PRALINE* program.

### Dabigatran etexilate -aptamers binding assays and dissociation constants (Kd) determination

For the binding assays, fluorescein labelled aptamers were synthesized. The K_ds_ of the selected aptamers were determined by performing binding assays. Each fluorescein-labeled sequence was heated to 90 °C for 10 min, 4 °C for 10 min and then 25 °C for 5 min. Different concentrations of labelled aptamers were incubated with dabigatran etexcilate beads for 1 hour. The beads were then washed with binding buffer and the bound DNA was eluted. The amount of DNA eluted from each sample was determined by fluorescence measurement. A saturation curve was obtained for each sequence and the dissociation constant for each aptamer was calculated by nonlinear regression analysis using Prism software.

### Electrochemical aptasensor for detection of Dabigatran

Square-wave voltammetry (SWV) measurements were carried out using disposable screen-printed gold electrodes were purchased from Dropsens, Inc. (Asturias, Spain). The electrodes include a standard three-electrode configuration that composed of working electrode (Au), Au counter and silver reference electrodes. Thiol-modified aptamer was immobilized on the Au working electrode via self-assembly by incubation for 12 hours in water-saturated atmosphere. The electrodes were then washed with Tris buffer and blocked with 1 mM mercapto hexanol. The SWV measurements of the DNA-modified gold electrodes were conduct in 5 mM [Fe(CN)_6_]^3−/4−^ solution prepared in 10 mM PBS, pH 7.4. The parameters of SWV measurements were: pulse amplitude: 20 mV, frequency: 25 Hz, a potential step: −5 mV, interval time: 0.04 s and scan rate: 125 mV s^−1^.

## Conclusions

We were able to select aptamers to the anticoagulant drug Dabigatran execilite. This work reports the first oligonucleotide aptamers selected for dabigatran *Etexilate* with K_d_ in the nanomolar range. These aptamers can be used for both basic research and clinical purposes. The selected aptamers demonstrate an excellent alternative to the monoclonal antibodies or Fab production against dabigatran *Etexilate* which can be utilized in biosensors as well as the development of antidote for Dabigatran. Future work will focus on developing aptamers-based biosensors and testing their ability to inhibit Dabigatran *Etexilate* action *in vitro* and vivo condition. Moreover, we believe that truncation of the originally selected aptamer sequences to enhance the binding affinity for its target would extend their diagnostic and therapeutic potentials^32^. We also consider introducing of modifications to the selected aptamers to increase their *in vivo* stability.
